# Synergistically Modulating Conductive Filaments in Ion‐Based Memristors for Enhanced Analog In‐Memory Computing

**DOI:** 10.1002/advs.202309538

**Published:** 2024-03-15

**Authors:** Jinyong Wang, Yujing Ren, Ze Yang, Qiaoya Lv, Yu Zhang, Mingyue Zhang, Tiancheng Zhao, Deen Gu, Fucai Liu, Baoshan Tang, Weifeng Yang, Zhiqun Lin

**Affiliations:** ^1^ School of Optoelectronic Science and Engineering University of Electronic Science and Technology of China Chengdu 611731 P. R. China; ^2^ Department of Electrical and Computer Engineering National University of Singapore Singapore 117576 Singapore; ^3^ Department of Chemical and Biomolecular Engineering National University of Singapore Singapore 117585 Singapore; ^4^ Department of Microelectronics and Integrated Circuit School of Electronic Science and Engineering Xiamen University Xiamen 361005 P. R. China; ^5^ Department of Electronic Science and Technology Harbin Institute of Technology Harbin 150001 P. R. China

**Keywords:** in‐memory computing, ion migration, memristor, oxygen vacancies, tunable filaments

## Abstract

Memristors offer a promising solution to address the performance and energy challenges faced by conventional von Neumann computer systems. Yet, stochastic ion migration in conductive filament often leads to an undesired performance tradeoff between memory window, retention, and endurance. Herein, a robust memristor based on oxygen‐rich SnO2 nanoflowers switching medium, enabled by seed‐mediated wet chemistry, to overcome the ion migration issue for enhanced analog in‐memory computing is reported. Notably, the interplay between the oxygen vacancy (Vo) and Ag ions (Ag^+^) in the Ag/SnO_2_/p^++^‐Si memristor can efficiently modulate the formation and abruption of conductive filaments, thereby resulting in a high on/off ratio (>106), long memory retention (10‐year extrapolation), and low switching variability (SV = 6.85%). Multiple synaptic functions, such as paired‐pulse facilitation, long‐term potentiation/depression, and spike‐time dependent plasticity, are demonstrated. Finally, facilitated by the symmetric analog weight updating and multiple conductance states, a high image recognition accuracy of ≥ 91.39% is achieved, substantiating its feasibility for analog in‐memory computing. This study highlights the significance of synergistically modulating conductive filaments in optimizing performance trade‐offs, balancing memory window, retention, and endurance, which demonstrates techniques for regulating ion migration, rendering them a promising approach for enabling cutting‐edge neuromorphic applications.

## Graphical abstract

This study leverages the synergy of modulating conductive filament to render a significant breakthrough in memory window, extending up to 1 million, and retention for up to 10 years, fostering advanced paradigms in bionics. These results underscore the judicious regulation of ion migration as a viable route to high‐performance memristors for neuromorphic computing applications.

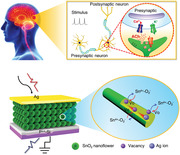

## 1 Introduction

The past decades have witnessed rapid advances in resistive switching (RS) devices, such as oxide‐based memristors, as the key enabler for next‐generation digital memory and in‐memory computing due to their versatile properties. These characteristics include non‐volatility, fast switching, low power consumption, compact device structure, and compatibility with complementary metal oxide semiconductor technologies.^[^ ^1^, ^2^ ^]^ Typically, the RS of memristors relies on the formation and rupture of conductive filaments in an amorphous medium, rendering data storage and processing in a single unit for energy‐efficient analog in‐memory computing.^[^ ^3^, ^4^, ^5^ ^]^ However, large‐scale implementation of these devices has consistently suffered from material‐level challenges. This is due to uncontrolled ion migration and stochastic filament location and morphology,^[^ ^6^ ^]^ thereby leading to poor spatial and temporal variations and limited optimization window between memory window, retention, and endurance.^[^ ^4^, ^7^, ^8^, ^9^ ^]^ In this context, the ability to engineer the filament topology and control the ion migration in active materials during RS represents an important endeavor for constructing robust memristors.

Depending on the types of mobile species involved in the formation and dissolution of conductive filament, the operation principles of memristors can be classified into cation‐based switching, anion‐based switching, and dual ionic switching.^[^ ^10^ ^]^ For cation‐based switching, RS materials typically contain reactive metal contacts (e.g., Ag^+^, Cu^+^, and Ni^2+^)^[^ ^11^ ^]^ that migrate under an applied electric field in the insulating matrix to form metallic filaments,^[^ ^12^, ^13^, ^14^ ^]^ offering excellent scaling potential, fast response, and low power operability.^[^ ^15^ ^]^ However, unstable filaments due to the high diffusivity of metal atoms/cations significantly compromise device performance, leading to high variability, poor endurance, and limited retention.^[^ ^14^, ^16^, ^17^ ^]^ In contrast, anion‐based resistive switching, characterized by the movement of negatively charged ions,^[^ ^14^, ^17^, ^18^ ^]^ such as dichalcogenide vacancies in transition metal dichalcogenides and oxygen vacancies (V_O_) in HfO_2_, holds the promise for enhanced endurance and retention.^[^ ^8^, ^19^ ^]^ While the migration of vacancies causes atomic structure changes in switching dielectrics, anionic RS devices typically necessitate an electroforming process to introduce soft breakdown of dielectrics at a high voltage. This unavoidably increases power consumption and device variability. Expanding on the concept of anion‐based and cation‐based switching, a related avenue involves dual ionic switching devices.^[^ ^20^, ^21^ ^]^ These devices combine both the cations and anions motion in the switching process, such as Ta ions and V_O_ in Ta/HfO_2_/Pt RRAM.^[^ ^20^ ^]^ The introduction of cations ions into the sub‐stoichiometric insulation medium with abundant vacancies could offer better control for modulating the dynamic switching process.

In addition to the mobile species, the microscopic structure of the host lattice also plays a crucial role in the RS characteristics.^[^ ^22^ ^]^ On the one hand, the anionic defects formation energy is significantly affected by their local atomic environment in the lattice. For instance, the presence of grain boundaries reduces the defect formation enthalpy, allowing the formation of anionic defects at more relaxed voltage biases than their perfect crystal.^[^ ^23^, ^24^ ^]^ On the other hand, the grain boundary serves as a 1D channel that effectively confines the percolation path of mobile ions and thus guides the growth/rupture of conductive filaments, achieving improved switching uniformity and reduced stochasticity.^[^ ^17^ ^]^ As such, the ability to incorporate V_O_ and active metal ions into one device, in conjunction with grain boundary engineering at the atomic level, offers a promising means of addressing the memristor's challenges.

Herein, we report a synergistic modulation of conductive filaments in ion‐based memristors for enhanced analog in‐memory computing, imparted by a reliable and scalable wet‐chemistry route to defective SnO_2_ nanoflowers (NFs) containing ample grain boundaries and V_O_ via temperature control. Specifically, SnO_2_ NFs are uniformly deposited onto a Si wafer to form a continuous thin‐film network. Subsequently, Ag metal is sputtered as a reactive top contact to yield the Ag/SnO_2_/p^++^‐Si memristor. Notably, the introduction of grain boundaries in SnO_2_ confines the diffusion of Ag^+^ within the 1D channel, significantly enhancing the switching uniformity. An in‐depth investigation reveals that the synergy between Ag^+^ and Vo in the Ag/SnO_2_/p^++^‐Si device renders effective modulation of the ion migration barrier in conductive filaments, resulting in a switching ratio of up to 10^6^ with low set/reset voltage variations. Furthermore, the Ag/SnO_2_/p^++^‐Si Resistive Random‐Access Memory (RRAM) manifests multiple synaptic functions, including paired‐pulse facilitation (PPF), long‐term potentiation (LTP), long‐term depression (LTD), and spike‐time dependent plasticity (STDP). The convolutional neural network based on the Ag/SnO_2_/p^++^‐Si RRAM achieves over 91.39% online learning accuracy for the Modified National Institute of Standard and Technology (MNIST) recognition tasks, enabled by the symmetric analog weight updating, thus demonstrating its capability for analog in‐memory computing.

## 2 Results and Discussion

### 2.1 Rational Design and Synthesis of Defective SnO_2_ NFs

**Figure 1:**
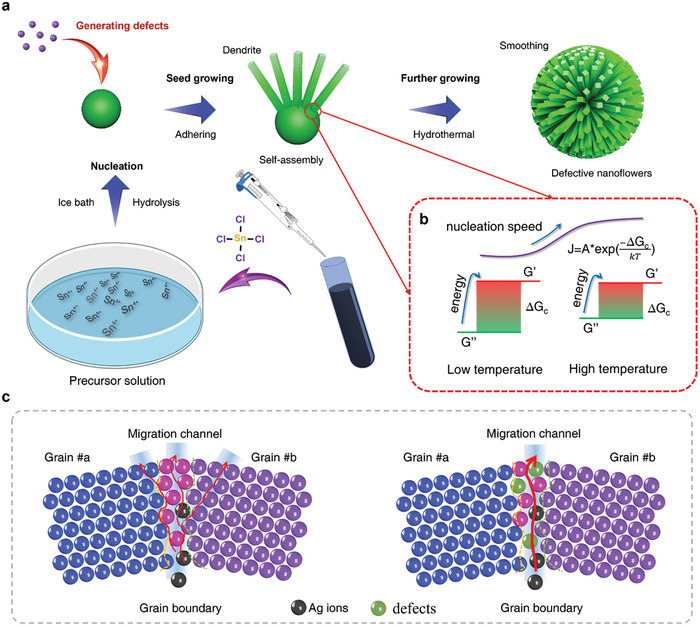
Schematic illustration of the synthesis of defective SnO_2_ NFs. a) Stepwise representation of the synthesis of SnO_2_ NFs, and b) the Gibbs theory‐based growth model for SnO_2_. c) The modulation of Ag ion and defect migration at grain boundaries.

To enable the modulation of ion migration kinetics in conductive filaments, we utilized a novel seed‐mediated wet‐chemistry approach to systematically design the defect states and microscopic structures of SnO_2_ NFs. As depicted in **Figure** **1a**, during the early stage of seed maturation, controlled dissociative Sn^4+^ ions in a 0 °C ice bath were used to modulate nuclei density and facilitate the formation of shallow defects at the surfaces surrounding the SnO_2_ seeds (Figure S1, Supporting Information).^[^ ^25^ ^]^ Figure 1b illustrates the SnO_2_ seed development model according to Gibbs theory. In equilibrium, density fluctuations that deviate from equilibrium occur in small regions of the system due to thermal fluctuations. During these fluctuations, atomic clusters (referred to as crystal nuclei) briefly emerge from Sn^4+^ ions, only to subsequently disperse and revert to their original state. Under supersaturated or supercooled metastable conditions, these variations expedite the transition from a monodisperse state to a self‐assembly state through thermodynamic and entropic processes in pursuit of achieving a Sn^4+^ state of maximum stability (Note I, Supporting Information).^[^ ^26^ ^]^ Non‐equilibrium defects on the surface of the SnO_2_ nanocrystalline layer trigger an increase in the number of grain boundaries, where abundant defective Vo accumulate. Subsequently, the activation energy for the diffusion of Sn^4+^ atoms reduces, thereby increasing the diffusion coefficient of Sn^4+^ and accelerating its diffusion, which introduces even more defective Vo by the reaction equation.^[^ ^27^ ^]^ Moreover, grain boundaries, considered bulk defects, create high or low‐energy surfaces by stabilizing dislocations that serve as ion migration channels due to lattice strain induced by the electric field. Oxygen vacancies are used to tailor the band structure and enhance the adsorption ability of reactants or intermediates.

(1) $$Sn^{4+}+\mathrm{Vo}+O_{2}⇌Sn^{4+}-{O_{2}}^{-}$$

Notably, the nucleation probability is highly dependent on the saturation of the system. Since the reaction happens in a supercooled metastable state (0 °C ice bath), the critical radius for SnO_2_ nuclei formation is greatly reduced. Thus, abundant nano‐sized SnO_2_ seeds form from the SnCl_4_ solution. While those SnO_2_ are defective due to non‐equilibrium reaction conditions where a large number of defective Vo are located near the SnO_2_ nanocrystalline surface and grain boundaries (Figure S2, Supporting Information). After the formation of defective SnO_2_ crystal nuclei, SnO_2_ seeds were further synthesized via a hydrothermal process, followed by thermal annealing under an N_2_ atmosphere to form the NFs morphology and crystallize the SnO_2_ NFs with abundant Vo for successfully designing vacancy‐rich SnO_2_ NFs based memristor. More comprehensive details regarding the preparation procedure can be found in the Experimental Section.

Sol‐gel processing techniques can yield nanocrystalline materials with the ability to finely passivate or modify grain boundaries, resulting in the alteration of grain boundary states.^[^ ^23^, ^28^ ^]^ By adjusting the reaction conditions at low temperatures during growth, it is possible to promote the preferential growth of specific grains or phases to bring about controlled grain boundary orientations of SnO_2_ NFs. Numerous grain boundary defects arise along diverse crystallographic orientations, as shown in Figure 1c and Figure S2 (Supporting Information). These defects create preferential pathways along the grain boundaries, guiding the movement of Ag ions and reducing their diffusivity in other directions, forming 1D migration channels. Consequently, Ag ion migration becomes more confined and controlled within these channels, leading to improved stability during the migration process. The presence of grain boundary defects enhances the overall efficiency of Ag migration, minimizing random diffusion and promoting more predictable and reliable behavior (Figure 1c).^[^ ^29^ ^]^ This controlled migration in 1D channels is advantageous for achieving stable SnO_2_ NFs memristors for advanced analog in‐memory computing, where precise and stable ion migration is essential for consistent and long‐lasting device performance.^[^ ^5^, ^30^ ^]^

**Figure 2:**
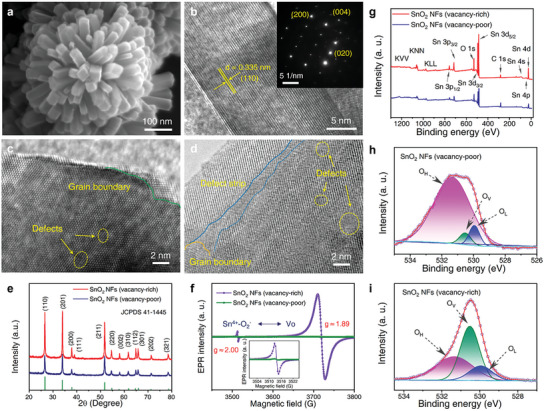
Characterization of the as‐synthesized defective SnO_2_ NFs. a) SEM image, b) high‐resolution TEM image and a SAED pattern (inset), c) and d) HRTEM images with heterophase grain boundaries, e) XRD patterns, f) EPR spectra, and g‐i) XPS spectra of full scanning g) and the O 1s h) and i) spectra with vacancy‐poor and vacancy‐rich, respectively.

Scanning electron microscopy (SEM) shows that the as‐synthesized SnO_2_ NFs were uniformly assembled on the substrate surface by radially distributed 1D rectangular nanorods (**Figure** **2**a; Figure S3a, Supporting Information). The transmission electron microscopy (TEM) images of the hierarchical nano‐pillar responsible for the formation of nanoflowers further characterize the structural feature and axial expansion along the (110) planes parallel to the SnO_2_ crystal structures (Figure S3b, Supporting Information). Lattice fringes with interlayer distance of 0.335 nm, corresponding to the (110) plane of rutile type of SnO_2_, were identified in the TEM (Figure 2b). The outermost surface of the SnO_2_ nanorod exhibits crystal facets aligned along the (110) plane, whereas the (001) plane is positioned perpendicular to the axis of the nanorod, indicating that the growth process was facilitated in the [001] direction.^[^ ^31^ ^]^ As shown in the inset of Figure 2b, point‐like patterns observed in the selected area electron diffraction (SAED) of a single SnO_2_ nanorod can be assigned to (200), (004), and (020) planes of rutile SnO_2_. Figure 2c,d illustrate grain boundary defects, playing a crucial role in restricting Ag ion migration to 1D channels, significantly impacting the migration process and stability. The X‐ray diffraction patterns of the synthesized SnO_2_ seeds, characterized by vacancy‐rich and vacancy‐poor, exhibit weak diffraction peaks at 26.61°, 33.89°, 51.78°, and 64.71° corresponding to the (110), (201), (211) and (112) planes of rutile SnO_2_ (Figure S4, Supporting Information). Notably, a slight peak shift was observed for vacancy‐rich SnO_2_ seed compared with vacancy‐poor SnO_2_ seed, indicating an enlarged lattice space due to the introduction of oxygen vacancies.

The XRD pattern of the SnO_2_ NFs with vacancy‐rich and vacancy‐poor indicates the presence of clear diffraction peaks that can be indexed to tetragonal rutile SnO_2_ (JCPDS41‐1445), thus demonstrating their high crystallinity (Figure 2e). In addition, electron paramagnetic resonance (EPR) spectroscopy was employed to analyze our defect‐related design strategy.^[^ ^25^ ^]^ The EPR spectrum of the vacancy‐poor SnO_2_ NFs shows negligible signals at g ≈1.89 and g ≈2.00. However, a strong and symmetric resonance peak was observed at the magnetic field of g ≈1.89 in vacancy‐rich SnO_2_ NF, indicating the presence of abundant V_O_. A prominent resonance peak at g ≈2.00 was detected, signifying the presence of a Sn^4+^‐O_2_^−.^ composite defect, forming a combination of super free radicals (Figure 2f). Furthermore, the Figure 2g displays the survey X‐ray photoelectron spectroscopy (XPS) spectra, revealing distinct signals corresponding to Sn, O, and C in samples with varying vacancy densities. The O 1s XPS spectra can be resolved into three peaks located at 531.35, 530.53, and 529.95 eV, attributing to hydroxyl oxygen (O_H_), oxygen vacancies (O_V_) and lattice oxygen (O_L_), respectively (Figure 2h,i; Figure S5, Supporting Information).^[^ ^32^ ^]^ Based on the fitting curve, the relative contents revealed the artful existence of O_H_, O_V_, and O_L_ of the O, with the proportion of O_V_ as high as 41.25%, indicating the presence of a large number of Vo defects (Table S1, Supporting Information).^[^ ^33^ ^]^ Using the wet chemistry temperature control method, vacancy levels in SnO_2_ NFs have been enhanced from 4.92% in SnO_2_ NFs with low vacancy levels to 41.25% in SnO_2_ NFs with high vacancy levels, resulting in a significant 7.38‐fold augmentation, which validates the effectiveness of the approach to material design. The results obtained confirm that the synthesis of SnO_2_ NFs results in the formation of abundant oxygen defects, providing the basis for an in‐depth analysis of their impact on filament memristor and ion synergistic properties.

### 2.2 Mechanism in Tunable Filaments with Enhanced Efficiency

**Figure 3:**
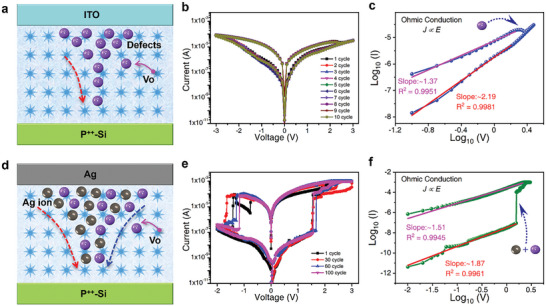
Comparison of *I‐V* characteristics and memristive conduction mechanisms between the ITO/SnO_2_/p^++^‐Si and the Ag/SnO_2_/p^++^‐Si devices. a) and d) The models for ITO/SnO_2_/p^++^‐Si and Ag/SnO_2_/p^++^‐Si devices, respectively. b) and e) *I–V* characteristics, and c) and f) Ohmic conduction supplemented by a double‐logarithmic depiction of *I–V* characteristics for the ITO/SnO_2_/p^++^‐Si and the Ag/SnO_2_/p^++^‐Si devices, respectively.

The synergistic contributions of Ag ions and oxygen vacancy defects were comparatively investigated in Ag/SnO_2_/p^++^‐Si and ITO/SnO_2_/p^++^‐Si devices (Figure 3d,e, respectively) to discuss the corresponding conduction mechanism.^[^ ^2^ ^]^ The process for preparing the device is elucidated in Figure S6 (Supporting Information), while the regular array scanning electron microscope (SEM) images and cross‐sectional views are shown in Figures S7,S8 (Supporting Information), respectively. *I–V* measurements were performed on the ITO/SnO_2_/p^++^‐Si device by tens of parallel voltage sweeps, as shown in **Figure** **3** **a,b**, where the current during voltage sweeps is lower than 10^−4^ A at 3 V (Figure S9, Supporting Information). In comparison, the Ag/SnO_2_/p^++^‐Si exhibits a large memory window of over 10.^6^ To investigate the origin of this performance discrepancy, the conduction mechanisms in both Ag/SnO_2_/p^++^‐Si and ITO/SnO_2_/p^++^‐Si devices were analyzed, respectively. The *I–V* curves are re‐plotted in Figure 3c,f in a double logarithmic scale. The power law dependence (I ∝*V^m^*) can be observed in a high resistance state (HRS) with slope variation in different electric field regimes showing that the HRS state of both devices follows trap‐associated space‐charge limited conduction (SCLC) theory. In contrast, the low resistance state (LRS) state is governed by ohmic conduction behavior, which is caused by the formation of conductive filament. Under enough electrical activation energy, electrons from oxygen vacancy defects could overcome the energy gap across the defective electrolyte, allowing themselves to transmit electricity inside the electrolyte and showing an Ohmic conduction model.

After replacing the TE with Ag, a noticeable resistive switching effect was observed in Ag/SnO_2_/p^++^‐Si (SnO_2_ NFs with vacancy‐rich) device where the device set and reset at lower voltages of 1.6 and −1.6 V, respectively, with an excellent memory window up to 10^6^. To demonstrate the synergistic effect of Ag ions and Vo vacancies, we devised an experimental setup consisting of Ag/SnO_2_/p^++^‐Si (SnO_2_ NFs with vacancy‐poor). This configuration was chosen to achieve on‐off ratios (≈600) that exhibit less than the on‐off ratios (1.29×10^6^) of Ag/SnO_2_/p^++^‐Si (SnO_2_ NFs with vacancy‐rich), as depicted in Figure 3e and Figure S10 (Supporting Information) respectively. In the reported research^[^ ^34^ ^]^ the switching ratio with Ag ions migration using the same material system with free defects was much lower than that in the Ag/SnO_2_/p^++^‐Si device in this work, which could also help to understand the synergistic activity of both Ag^+^ cations and oxygen vacancy defects. Following formation, the Ag/SnO_2_/p^++^‐Si device presents a LRS region. When operated at less than 1.6 V, the Ag/SnO_2_/p^++^‐Si device was switched to an HRS prior to initiating the formation of the filaments, shedding light on the operational behavior between voltage dynamics and filamentary processes within the device. Figure 3f shows the double logarithmic plot of the *I‐V* curve for the Ag/SnO_2_/p^++^‐Si device. The Ohmic conduction mechanism of *J∝E* is demonstrated to be dominant inside the Ag/SnO_2_/p^++^‐Si device when subjected to an excessive applied voltage, as evidenced by the R^2^ values of 0.99 and slopes of 1.87 in the double‐logarithmic *I–V* curves. This observation implies that the oxygen vacancy defects and the Ag^+^ cations, while aligned parallel to the platform, collaborate to form charge‐transfer channels, which facilitate the migration of Ag ions and vacancies and serve as electronic hopping points when an electrical voltage is applied. Consequently, in combination with *I–V* test results, it is inferred that oxygen vacancy defects could influence the conductivity, albeit to a lesser extent. In accordance with the valence change process, the conductivity of Ag ions increases as the electrotherapy electricity increases because more Ag ions migrate through the vertically stacked electrolytic oxide layers with SnO_2_ NFs.^[^ ^12^ ^]^ Therefore, electrical stimulation could be used to linearly regulate the conductance in the Ag/SnO_2_/p^++^‐Si memristors.

**Figure 4:**
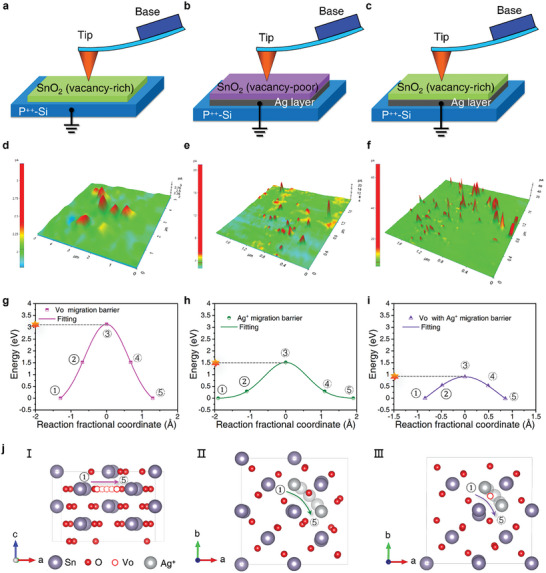
C‐AFM mappings and DFT calculations. C‐AFM analysis was conducted with the device structures of a) SnO_2_ (vacancy‐rich)/P^++^‐Si, b) SnO_2_ (vacancy‐poor)/Ag/P^++^‐Si, and c) SnO_2_ (vacancy‐rich)/Ag/P^++^‐Si. The corresponding (a) to c)) C‐AFM mappings are shown in d) to f). Comparison of the migration energy of g) Vo, h) Ag^+^, and i) Vo with Ag^+^. j) The diffusions along the respective pathways in g‐i) are represented by I, II, and III. The yellow dots on the plots of migration energy g‐i) illustrate which visualizations were used in the nudged elastic band analysis.

We designed four device structures and validated the synergistic interaction between Ag^+^ ions and oxygen vacancies through the conducting atomic force microscope (C‐AFM) (**Figure** **4a–f**; Figure S11, Supporting Information). A voltage bias of 2 V is applied to the C‐AFM tips, while the sample is grounded in contact mode with similar conductivity baselines. For SnO_2_ NFs (vacancy‐poor) on inert p^++^‐Si, nanofilament density and strength are very low compared with SnO_2_ NFs (vacancy‐rich) shown in Figure 4a,d and Figure S12 (Supporting Information). After replacing the P^++^‐Si with Ag, a tenfold increase in the current from 3 to 20 pA was observed in SnO_2_ (vacancy‐poor)/Ag/P^++^‐Si (Figure 4b,e). This means that the presence of abundant Vo states favors the formation of conductive filaments in SnO_2_ NFs. Notably, when silver ions (Ag ions) coexist with abundant Vo, the strength of the conductive filament produced becomes higher to 60 pA (Figure 4c,f), highlighting the synergistic effect between Ag ions and oxygen vacancy defects. The comparison results demonstrate that the interplay between oxygen vacancies and Ag ions (Ag^+^) cations in the Ag/SnO_2_/p^++^‐Si memristor efficiently modulates the formation and abruption of conductive filaments.

We employed the Climbing Image Nudged Elastic Band (CI‐NEB) calculation method to determine the migration barrier of filament paths, enabling us to understand better the mechanisms of filament formation and disruption and design materials with low migration barriers.^[^ ^35^ ^]^ Our calculation model was based on SnO_2_ XRD findings, which were optimized for good matching (Table S2, Supporting Information). Figure 4g–i depicts the minimum energy pathway for two in‐plane and two cross‐plane diffusion channels involving Vo, Ag^+^, and Vo with Ag^+^. All minimum energy pathways we explored were nearly symmetric and included a single transition state. The diffusion of Vo, Ag^+^, and Vo with Ag^+^ along pathways ① and ⑤ occurs within the same plane, and Figure 4j (I, II, and III) shows that the possible destinations for ① and ⑤ lie within the same coordination polyhedron. For in‐plane diffusion, Ag^+^ diffusion to a Vo vacancy in the same coordination polyhedron (site ①) is preferred over diffusion to a Vo vacancy in a neighboring polyhedron (site ⑤), consistent with previous DFT investigations.^[^ ^36^ ^]^ The synergy between ① and ⑤ in creating conductive filaments is further demonstrated by a significant reduction in the migration barrier when placed in the migration route of Vo with Ag^+^ due to the most favorable coordination polyhedron. Our devices and CI‐NEB calculations demonstrated that Ag^+^ migration and the synergy effect between Ag^+^ and Vo in the Ag/SnO_2_/p^++^‐Si device can effectively modulate conductive filaments, resulting in a high switching ratio.

### 2.3 Resistive Switching and Synaptic Performance

**Figure 5:**
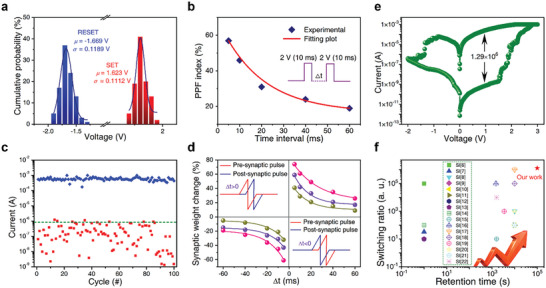
Memristive and synaptic characteristics of Ag/SnO_2_/p^++^‐Si device. a) Cumulative probability distribution of setting and resetting voltages with 1 µA *I*_cc_. b) PPF. c) Variation in resistances across 100 consecutive set/reset cycles. d) Adjustment of synaptic weight (△W) regulated by pulses with varying lead/lag times (△t) and voltage (pulse with a pulse amplitude of ±1.8, ±2.0, and ±2.2 V, pulse duration of 500 ns, and rise time of 10 ns). e) The *I–V* characteristic and f) switch ratio and retention time compared with the reported references.

The resistive switching performance of Ag/SnO_2_/p^++^‐Si devices was evaluated. Typical *I–V* curves were generated from memristor devices with scan ranges of −1.5 V to 3 V and ‐2 V to 3 V, holding for 100 cycles (Figures S13–S16, Supporting Information), showing nonvolatile switching characteristics. The starting resistance of the device was above 50 GΩ, and the biased voltages were swept as follows: 0 V → 3 V → 0 V → 2 V → 0 V. Using a hundred *I–V* charts, the absolute value of the standard deviation (*σ*) divided by the mean value (*µ*) for the SET and RESET voltages were obtained as shown in **Figure** **5a**. The current/voltage variation within a device can be attributed to the random behavior of filaments during the bipolar resistive switching operation. At *I_cc_* = 0.1 µA, the SET and RESET voltages were *µ* = 1.623 V (*σ* = 0.1112 and switching variability, *S_V_* = 6.853%) and *µ =* −1.669 V (*σ* = 0.1189 and *S_V_* = −7.1244%), respectively.^[^ ^37^ ^]^ Figures S17,S18 (Supporting Information) illustrate the retention and endurance performance test results of the memristor utilizing SnO_2_ NFs. Retention testing, in accordance with the Arrhenius relation, was conducted at 175, 210, 245, and 280 °C. ^[^ ^38^, ^39^ ^]^ A retention of 8.4869×10^8^ s at 85 °C (an industry standard)^[^ ^39^ ^]^ was extrapolated from the linear fit of the measurement results, surpassing the threshold for a 10‐year duration.^[^ ^40^ ^]^

As in physiological neurons, the synaptic weight of artificial synaptic memristors can also be modulated by the time difference between the initial (A_1_) and subsequent (A_2_) serial pulses, a phenomenon called paired‐pulse facilitation (PPF) and belongs to a type of short‐term plasticity (STP) (Figure S19, Supporting Information).^[^ ^41^ ^]^ Here, the Ag/SnO_2_/p^++^‐Si device was simulated by altering the duration (△t) with 10 ms at a biased voltage of 3 V between the two positive pulses, and the below formula was used to determine the PPF index:^[^ ^42^ ^]^

(2) $$\mathrm{PPF}\;\mathrm{index}=\left(A_{2}-A_{1}\right)/A_{1}\times 100\%$$

where A_1_ and A_2_ represent the first and second excitatory postsynaptic currents (EPSC) values produced by two sequential voltage pulses. The empirically determined PPF index was fitted using the following exponential equation:

(3) $$Y=1+\left\{C_{1}\times \exp\left(-\Delta t/\tau_{1}\right)+C_{2}\times \exp\left(-\Delta t/\tau_{2}\right)\right\}$$

where C_1_ and C_2_ are the amounts of the initial relaxing, and *τ*_1_ and *τ*_2_ are the usual easing durations for the fast and slow decaying factors (Figure 5b). The observed fluctuation in relaxation durations is apparently in agreement with the functioning of physiological interactions among neurons, suggesting that our Ag/SnO_2_/p^++^‐Si memristor devices could be utilized to replicate natural synaptic short‐term plasticity (STP) properties. To acquire the SET and RESET switching properties, the *I–V* curves were measured over a period of more than a hundred times. It is observed that the obtained *V_SET_* and *V_RESET_* exhibit limited changes, and the resistance values in the LRS are highly consistent throughout the hundred cycles (Figure 5c).

In addition, the duration of both pre‐ and post‐synaptic spikes has a significant impact on the synaptic weight fluctuations, the phenomenon called spike‐time‐dependent‐plasticity (STDP).^[^ ^43^ ^]^ The effective demonstration of STDP is crucial to establish the validity of the produced electronic synapses for artificial neural systems since it is one of the fundamental Hebbian learning principles of biological synapses.^[^ ^9^ ^]^ To illustrate the STDP behavior of our Ag/SnO_2_/p^++^‐Si memristor device, we have developed a pulse pattern that is optimal for this function. In this situation, presynaptic stimulating is activated by Ag TE, whereas postsynaptic stimulating is activated by the p^++^‐Si bottom electrode (BE). A long‐term potentiation (LTP) will occur as a consequence of this situation if the presynaptic pulse arrives before the postsynaptic pulse (△t > 0). On the other hand, long‐term depression (LTD) will occur if the postsynaptic pulse arrives before the presynaptic pulse (△t > 0). The equation △t = t_pre‐pulse_‐t_post‐pulse_ could be used to determine the difference in pulse time in both the pre‐synaptic and post‐synaptic periods. A graphic depiction of pre‐synaptic and post‐synaptic pulses is illustrated in Figure 5d, and the associated pulse properties are determined as below:^[^ ^44^ ^]^

(4) $$\Delta W=A^{+}\mathrm{ex}p^{\frac{-\Delta \tau }{\tau^{+}}}\left(t>0\right)$$

(5) $$\Delta W\;=\;A\;\mathrm{ex}p^{\frac{-▵t}{\tau^{-}}\;}\;\left(t\;<\;0\right)$$

where A^+^ and A^−^ are scaling factors, τ^+^ and τ^−^ are decay constants. Once the temporal variation (△t) is greater than zero, the synaptic weight shift (△W) increases with the decrease in △t, suggesting the existence of the LTP function. When △t is less than zero, on the other hand, △W decreases with a rise in △t, demonstrating the presence of the LTD function. Therefore, a greater activation of the device conductance shifts with a smaller △t between the stimulating pulses because of a more significant increase in synaptic weight. These findings confirm the STDP classification model with tunable variation in synaptic weight in our Ag/SnO_2_/p^++^‐Si memristor devices, which successfully mimics the behaviors of biological synapses. In addition, our investigation reveals that the statistically determined switch ratio of SnO_2_‐based memristor, coupled with the switch ratio reported in the references (Figure 5e,f and Table S4, Supporting Information), underscores the considerable potential of synergistically modulating conductive filaments in ion‐based memristors for advancing analog in‐memory computing.

### 2.4 Implementation of Image Recognition and Hardware MAC Operation

**Figure 6:**
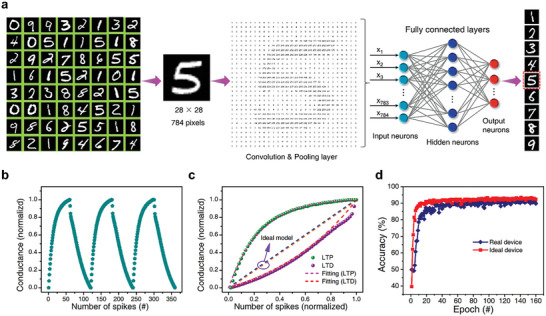
Implementation for image recognition based on Ag/SnO_2_/p^++^‐Si devices. a) A digital information schematic depicting the steps taken by a neural network during deep learning training using the Modified National Institute of Standards and Technology (MNIST) dataset. b) Neuronal plasticity and the reprogramming of synaptic conductance states. Memristors have pre‐programmed pulse voltages that may be up to 360 times the normal voltage. (60 pulses at +1.2 V for 15 ms and 60 pulses at −1.2 V for 15 ms). c) Extremely low noise and a customizable synaptic conductance of 3 cycles. d) Epoch‐based accuracy in image identification.

A low‐noise, multi‐stage neuromorphic computing system is entirely feasible by utilizing recorded synaptic properties and stochastic gradient descent to construct a 3‐layer deep neural network training with back‐propagation.^[^ ^45^ ^]^ To demonstrate this, we designed a network architecture based on Ag/SnO_2_/p^++^‐Si memristor devices to recognize images from the MNIST,^[^ ^46^ ^]^ which contains 28×28 pixel images of handwritten digits. The Convolutional Neural Network (CNN) network was constructed by a passively sandwiched array hardware design that consists of a single structure of Ag/SnO_2_/p^++^‐Si, with 784 input units, 300 neurons in the hidden layer, and ten output neurons (**Figure** **6a**). The empirically recorded conductance states are utilized to obtain synaptic weight, improving the effective model of the frameworks (Figure 6b,c). The Ag/SnO_2_/p^++^‐Si memristors' conductance variations are shown to be very symmetric and linear, making them suitable for programming with voltage pulses by 360 times at lower noises.

The variations in conductance potentiation (△Gp) and depression (△Gd) could be described by the following formulas, which provide further evidence of the non‐linearity:

(6) $$\begin{array}{ccc} \Delta G_{p}:G_{n+1} & = & G_{n}+\Delta \;G_{p}\\ & = & G_{n}+\alpha_{p}\exp\left[-\left(G_{n}-G_{\min}\right)/k_{p}\left(G_{\max}-G_{\min}\right)\right] \end{array}$$

(7) $$\begin{array}{ccc} \Delta G_{d}:G_{n+1} & = & G_{n}+\Delta G_{d}\\ & = & G_{n}-\alpha_{d}\exp\left[-\left(G_{n}-G_{\min}\right)/k_{d}\left(G_{\max}-G_{\min}\right)\right] \end{array}$$

where k and α represent the quantity of nonlinearity and conductance change correspondingly. Compared to prior publications, the nonlinearity of potentiation (k_p_ = 0.12) and depression (k_d_ = 0.09) is astonishingly near zero (Table S3, Supporting Information).^[^ ^47^ ^]^ Utilizing these conductance differences as synaptic weights, a neural network based on Ag/SnO_2_/p^++^‐Si memristors achieved an accuracy of up to 91.39% on the MNIST identification task using ≈60 000 training and 10 000 test datasets (Figure 6d). The accuracy achieved, as low as 1.22%, is still below that of the theoretically ideal device, whose performance is limited to 3% in a floating‐point‐based neurological system. These impressive results can be attributed to the adjustable synaptic weights with low noise and good linearity, facilitated by efficient tunable filaments, which are boosted by Ag^+^‐cation migration and the synergy defects in Ag/SnO_2_/p^++^‐Si memristor devices.

**Figure 7:**
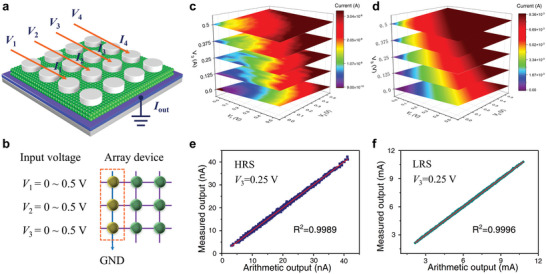
The Multiply‐accumulate (MAC) processing implementations using SnO_2_‐based memristive hardware. a) and b) The schematic representation of the memristor‐based array performing the MAC operation. Mapping of output current in the voltage‐dependent hardware array (*V*_3_). Three High‐resistance state (HRS) and Low‐resistance state (LRS) devices share a column (c and d). The input voltage for *V*_3_, with respect to *V*_1_ and *V*_2_, consists of 33 steps. e) The memristors in a column maintain a state of HRS and f) a state of LRS and linear fitting results, respectively.

Furthermore, we have incorporated multiply‐and‐accumulate (MAC) verification for hardware implementation.^[^ ^18^, ^48^, ^49^ ^]^ **Figure** **7** **a,b** implement a compact 3×3 array inside an array. The device's conductance distribution is highly uniform within a narrow area, leading to efficient MAC operations. The three devices in the same column are located at an HRS and LRS, respectively. It is important to note that only 5 mappings are displayed in Figure 7c,d. Nevertheless, the *V*_3_ input voltage also consists of 33 steps, similar to the *V*_1_ and *V*_2_ inputs. Achieved favorable output linearity in the HRS and LRS at *V*_3_ = 0.25 V, with R^2^_(HRS)_ = 0.9989 and R^2^_(LRS)_ = 0.9996 (Figure 7e,f), indicating good MAC operation results and showcasing the potential applicability to hardware implementation of the kernel image processing.

## 3 Conclusion

In summary, we developed a scalable sol‐gel technique to craft SnO_2_ NFs with abundant oxygen vacancies (Vo) as an effective accelerator for neuromorphic systems with, for the first time, a high resistive switching ratio. The Ag^+^ cations and Vo transport in the Ag/SnO_2_/p^++^‐Si RRAM synergistically modulated the conductive filaments, resulting in a switching ratio greater than 10^6^ with low set/reset voltage variabilities. By capitalizing on the CI‐NEB technique, the underlying mechanisms of the formation and disruption of Vo, Ag^+^, and Vo with Ag^+^ filament morphologies were revealed to provide insight into the development of materials with a low migration barrier. The resulting memristors could render multiple functions of synaptic plasticity, including PPF, LTP, LTD, and STDP. The symmetric analog weight updating, and various conductance states enable convolutional image processing with a recognition accuracy of ≥ 91.39%, demonstrating its capacity for high‐accuracy digital computing and energy‐efficient in‐memory computing. As such, our study emphasizes the importance of synergistically modulating conductive filaments to optimize performance trade‐offs, maintaining balance in memory window, retention, and endurance, which demonstrates techniques to regulate ion migration, making it a promising avenue in the realm of future advanced neuromorphic applications.

## 4 Experimental Section

### Material Preparation

The 100 µL of SnCl_4_ was injected into a 100 mL vial containing 50 mL of deionized (DI) water on ice, then vigorously agitated until the ice had melted. To produce the SnO_2_ seeds, a mixture of 10 mL SnCl_4_ solution and 5 mL DI water was stirred at 50 °C for 48 h in a water bath, and the precipitate was washed, dried at 65 °C, and annealed at 500 °C under a nitrogen atmosphere. The resulting SnO_2_ seeds were milled to fine powders and mixed with 0.028 g mL^−1^ NaOH solution, and 240 µL of SnCl_4_ was immediately added to the mixture, which was agitated for the SnO_2_ NFs with vacancy‐rich in an ice bath and the SnO_2_ NFs with vacancy‐poor in a 25 °C bath sustaining 30 min. The solution was then transferred to autoclaves and heated at 200 °C for 24 h. The final product was collected and air‐dried at 65 °C.

### Device Fabrication

To fabricate ITO/SnO_2_/p^++^‐Si or Ag/SnO_2_/p^++^‐Si memristor devices, P^++^‐Si was utilized as the substrate. The preparation process for Ag/SnO_2_/p++‐Si memristor devices for vacancy‐rich and vacancy‐poor conditions follows identical steps. First, a SnO_2_ film was deposited on the P^++^‐Si substrate by spin‐coating SnO_2_ NFs solution. The spin‐coating process was conducted at a low speed of 1000 rpm for 15 s, followed by a high rate of 3000 rpm for 45 s. Subsequently, Ag was sputtered onto the substrate using a shadow mask to form the top electrode (TE). Furthermore, ITO or Ag was deposited as a controlled sample for the TE.

### Characterization and Device Test System

The analysis utilized several techniques to characterize the SnO_2_ NFs. X‐ray diffraction (XRD) patterns were collected using Cu Kα radiation (λ = 0.15 418 nm) on an Ultima IV instrument (Rigaku, Japan). Electron paramagnetic resonance (EPR) spectroscopy was performed on a BRUKER A300 instrument at sub‐110 K. Field‐emission scanning electron microscope (FE‐SEM) images were obtained using an FEI Inspect F instrument at 20 kV. Transmission electron microscopy (TEM) images, elemental mapping, and corresponding selected area electron diffraction (SAED) patterns were obtained using a Tecnai G2 F20 S‐Twin TMP instrument at an acceleration voltage of 200 kV. The electrical measurements were carried out using a probe station equipped with a Keithly 2636B system.

### Statistical Analysis

To model the learning capabilities of neuromorphic computing, a neural network using backpropagation to update synaptic weight based on recovered device characteristics, including nonlinearity, asymmetry, and the number of effective conductance states was trained. The processed current obtained at the output neurons was computed using a matrix product of the input signal as voltage to the input neurons, which was then utilized to adjust synaptic weight changes in conductance. A fully connected neural network structure was adopted for the detection of MNIST handwritten digits with a resolution of 28×28 pixels.^[^ ^18^, ^48^ ^]^ The network consists of an input layer of 784 neurons representing the digits 0–9, a fully connected hidden layer of 300 neurons, and an output layer of 10 neurons. To switch on the neural network, the sigmoid function unit was utilized (y = 1/1+exp(‐x)). A total of 160 training iterations were carried out for neural networks. The entire network was implemented using the PyTorch framework and performed convolutional processing in the VS Code software. All experiments were conducted on GPUs (Nvidia GTX 1080Ti) to ensure efficient processing.

### Computational Method

The calculations presented in this analysis are based on Density functional theory (DFT) as implemented in the Vienna Ab initio Simulation Package (VASP) code, utilizing the projector augmented wave (PAW) method.^[^ ^50^ ^]^ The Perdew‐Burke‐Ernzerhof (PBE) form of the generalized gradient approximation (GGA) was utilized to provide the exchange and correlation functional.^[^ ^51^ ^]^ A cutoff energy of 400 eV was employed, and a dense Brillouin zone was computed using a 3×3×5 Monkhorst‐Pack k‐point sampling.^[^ ^52^ ^]^ Structural relaxation was terminated when all forces were less than 0.01 eV Å^−1^, and all bulk crystal structures were fully optimized. To determine the feasibility of different diffusion pathways, a (2×2×2) supercell was employed. The migration barriers of Ag interstitial and Vo vacancy were determined using the climbing image nudged elastic band (CI‐NEB) method.^[^ ^53^ ^]^ All calculations were performed using a 3×3×5 Monkhorst‐Pack grid, and the ground state configurations and CI‐NEB bands were minimized to less than 0.03 eV Å^−1^ of total forces on each ion.

## Conflict of Interest

The authors declare no conflict of interest.

## Supporting information

Supporting Information

## Data Availability

The data that support the findings of this study are available in the supplementary material of this article.
