# Supplementary material for: Synergistically Modulating Conductive Filaments in Ion‐Based Memristors for Enhanced Analog In‐Memory Computing
Source: Adv Sci (Weinh). 2024 Mar 15;11(22):2309538. doi: 10.1002/advs.202309538 (PMC11165545; doi:10.1002/advs.202309538)
Supplement: Supplementary file 1 — Supporting Information [file ADVS-11-2309538-s001.pdf]

ADVANCED SCIENCE
Open Access

Supporting Information

for *Adv. Sci.*, DOI 10.1002/advs.202309538

Synergistically Modulating Conductive Filaments in Ion-Based Memristors for Enhanced Analog In-Memory Computing

Jinyong Wang, Yujing Ren, Ze Yang, Qiaoya Lv, Yu Zhang, Mingyue Zhang, Tiancheng Zhao, Deen Gu, Fucai Liu, Baoshan Tang*, Weifeng Yang* and Zhiqun Lin**

WILEY-VCH

Copyright WILEY-VCH Verlag GmbH & Co. KGaA, 69469 Weinheim, Germany, 2023.

Supporting Information

Synergistically Modulating Conductive Filaments in Ion-Based Memristors for Enhanced Analog In-Memory Computing

Jinyong Wang‡*, Yujing Ren*‡*, Ze Yang, Qiaoya Lv, Yu Zhang, Mingyue Zhang, Tiancheng Zhao, Deen Gu, Fucai Liu***, Baoshan Tang***, Weifeng Yang***,* and *Zhiqun Lin**

Y. Ren, Dr. M. Zhang and Prof. Z. Lin

Department of Chemical and Biomolecular Engineering, National University of Singapore, Singapore 117585, Singapore

J. Y. Wang, T. C. Zhao, Prof. D. Gu and Prof. F. C. Liu

School of Optoelectronic Science and Engineering, University of Electronic Science and Technology of China, Chengdu 611731, PR China.

J. Wang, Q. Lv and Dr. B. Tang

Department of Electrical and Computer Engineering, National University of Singapore, Singapore 117576, Singapore

Z. Yang and W. Yang

Department of Microelectronics and Integrated Circuit, School of Electronic Science and Engineering, Xiamen University, Xiamen 361005, PR China

Y. Zhang

Department of Electronic Science and Technology, Harbin Institute of Technology, Harbin 150001, PR China

‡These authors contributed equally to this work (J. Wang and Y. Ren).

*Corresponding author. Email: fucailiu@uestc.edu.cn (F. Liu), baoshantang@nus.edu.sg (B. Tang), wfyang@xmu.edu.cn (W. Yang), z.lin@nus.edu.sg (Z. Lin).

Note I: SnO_2 NFs growth theory.

The Gibbs theory for the SnO_2 NFs process is analyzed as follows. The free energy change of the crystal nucleus system is:

$$\Delta G = \Delta G_s - \Delta G_v \qquad (1)$$

$$= -\frac{4}{3}(\Delta R^3 \Delta g/\Omega) + 4\pi R^2 \sigma$$

where ΔG represents the free energy change (negative value) of the system caused by the emergence of crystal nuclei, ΔG_v represents the free energy change per unit volume, Ω represents the volume of particles (specific volume), ΔG_s represents the free energy change of the additional surface (positive value) caused by the formation of new crystal faces, σ represents the specific surface energy.[1]

When R is small, ΔG_s plays a significant role and the free energy of the system increases; when R is high, ΔG_v plays an important part and the free energy of the system decreases. When the criterion $\frac{\partial \Delta G}{\partial R} = 0$ is achieved, G achieves its most significant value, and the critical radius of the crystal nucleus is calculated as

$$R_c = \frac{2\sigma\Omega}{\Delta g} = \frac{2\sigma\Omega}{kT \ln \alpha} \qquad (2)$$

The formula that incorporates the free energy of the crystal nucleus can be obtained as

$$\Delta G_c = \frac{16\pi\sigma^3\Omega^2}{3(\Delta g)^2} = \frac{4}{3}\pi\sigma R_c^2 \qquad (3)$$

where ΔG_c denotes the height of the barrier or the formation energy that must be surpassed to create critical nuclei in the system. When $R<R_c$, the probability of crystal nucleus ablation is more significant than that of growth; when $R>R_c$, the probability of crystal nucleus growth is greater than that of ablation. The critical cluster can become a crystal nucleus of a new phase, and the likelihood of development is more significant than that of dissolution.

From the critical radius and formation energy formula, it can be determined that the critical crystal nucleus is smaller, and its formation energy is lower than the more significant degree of

supersaturation of the solution.

The Arrhenius reaction rate equation[2] could be used to calculate the nucleation rate (J), which is defined as the number of crystal nuclei produced per unit of time and unit volume:[3]

$$J = A \exp(-\Delta G_c/kT) = A \exp\left[-\frac{16\pi\sigma^3\Omega^2}{3k^3T^3(\ln\ )^2}\right] \quad (4)$$

These all have a common pre-exponential component denoted by the letter A.

According to the calculation, T, σ and α are the primary factors impacting the nucleation rate. Based on the research mentioned above, the nucleation rate is very sensitive to changes in supersaturation. To accomplish the goal of regulating SnO_2 NFs growth, it is necessary, under the same experimental circumstances, to decrease the temperature but to increase the saturation of the system and the nucleation rate.[4]

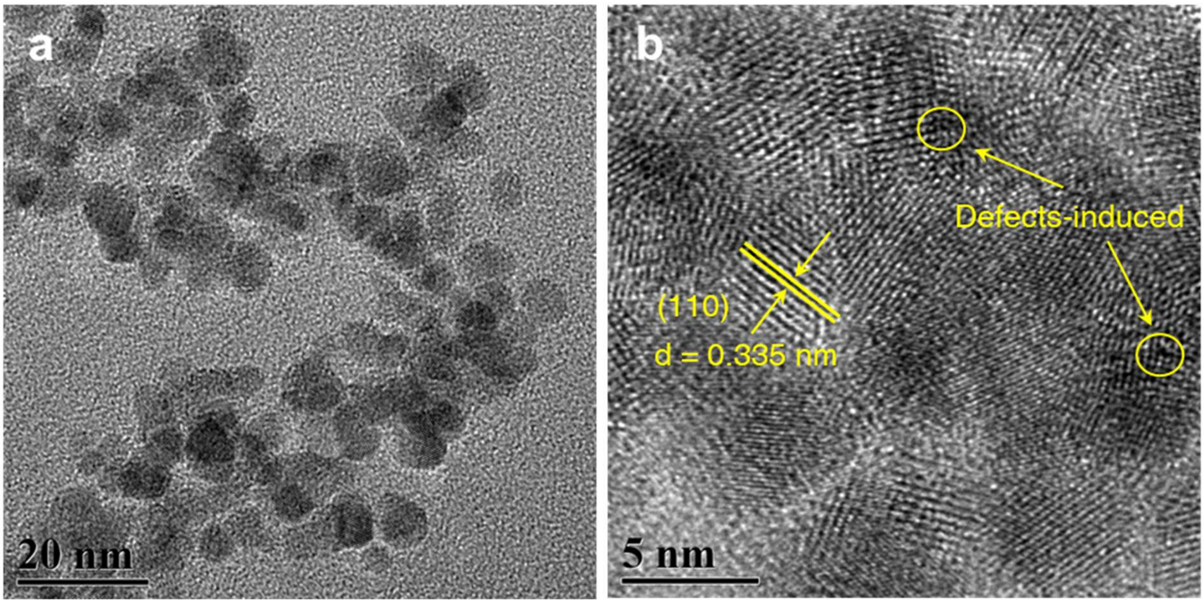

Figure S1. a) and b) HTEM images of SnO_2 NFs seeds.

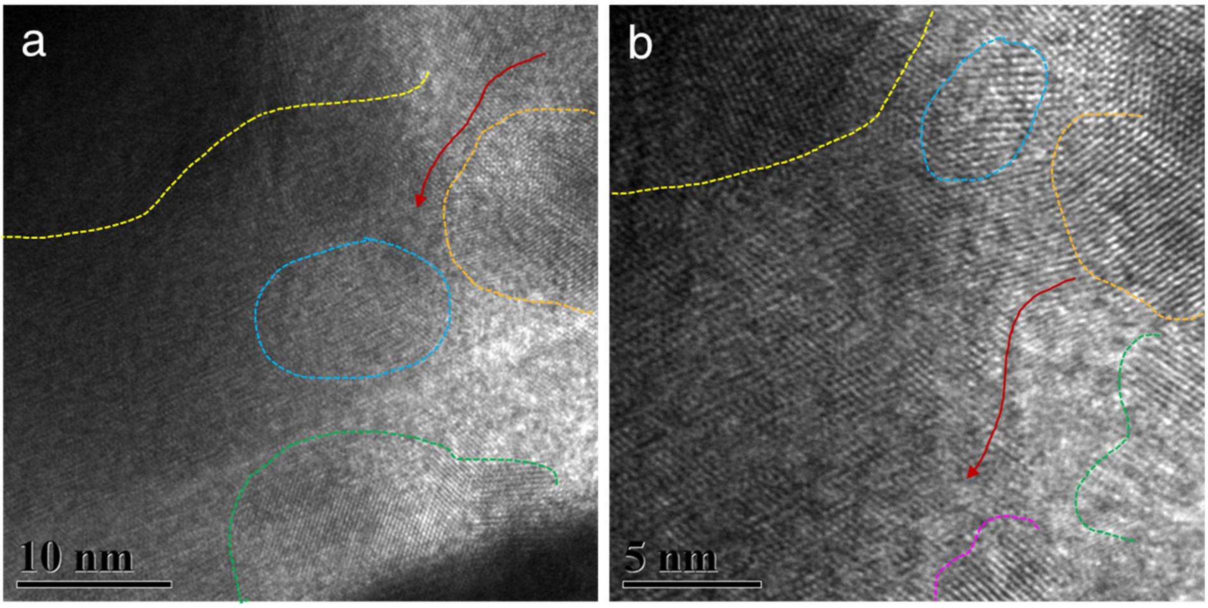

Figure S2. a) and b) HRTEM images with heterophase grain boundaries of SnO_2 NFs.

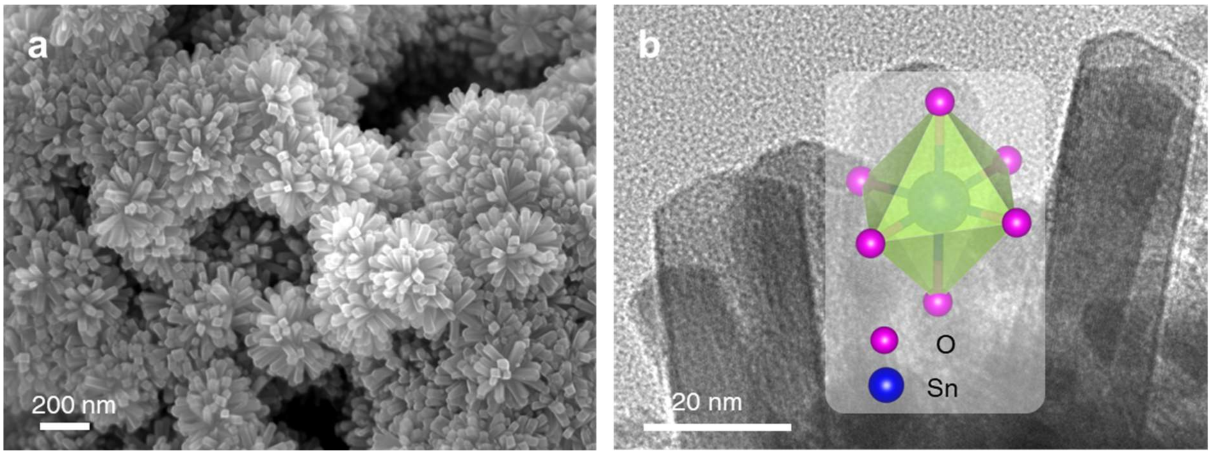

Figure S3. Characterization of the fabricated defective SnO_2 NFs a) SEM image, b) TEM image with a corresponding SnO_2 lattice unit (inset).

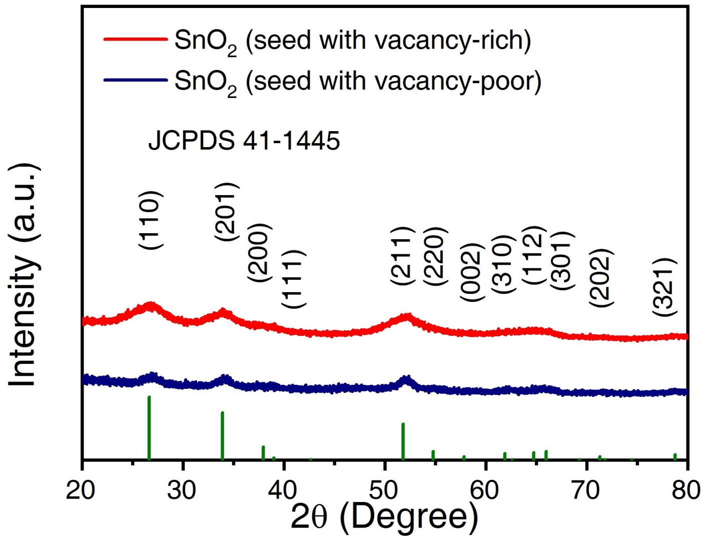

Figure S4. XRD peaks of SnO_2 seeds with vacancy-rich and vacancy-poor.

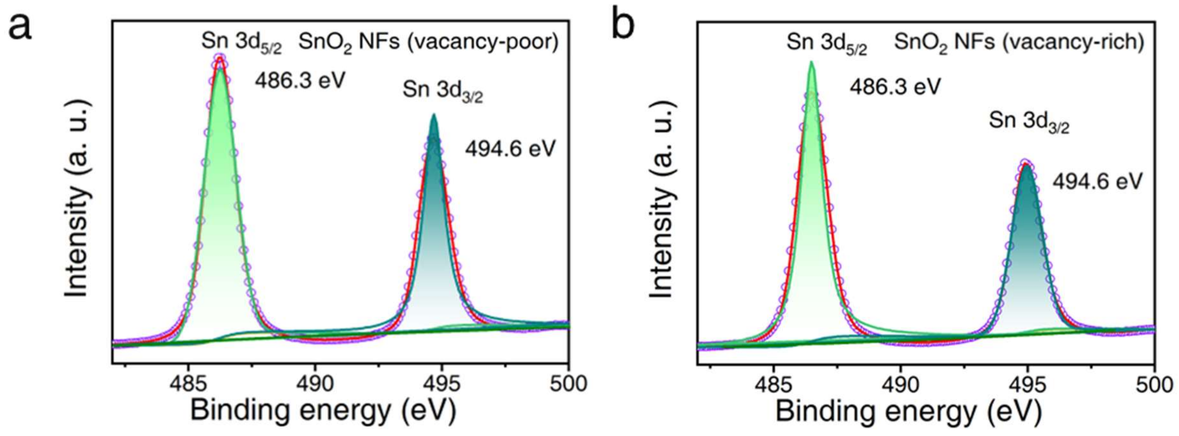

Figure S5. XPS spectra of the Sn 3d of a) vacancy-poor SnO_2 NFs and b) vacancy-rich SnO_2 NFs.

The Sn 3d orbital peaks in pure tin dioxide exhibit two binding energies at 486.3 and 494.6 eV for vacancy-poor SnO_2 NFs and vacancy-rich SnO_2 NFs, corresponding to the characteristic peaks of Sn^{4+} $3d_{5/2}$ and $3d_{3/2}$, respectively.[5] These observations demonstrate the presence of only one Sn species with a +4 valence state in SnO_2 NFs.

Table S1. The area of the different valence of O and content ratio of vacancy-poor SnO_2 NFs and vacancy-rich SnO_2 NFs.

Simples	Valence	Area	Content ratio (%)
SnO_2 NFs (vacancy-poor)	O_H	59608.43	87.37
	O_V	3360.39	4.92
	O_L	5255.25	7.71
SnO_2 NFs (vacancy-rich)	O_H	148613.50	39.08
	O_V	156836.00	41.25
	O_L	74778.46	19.67

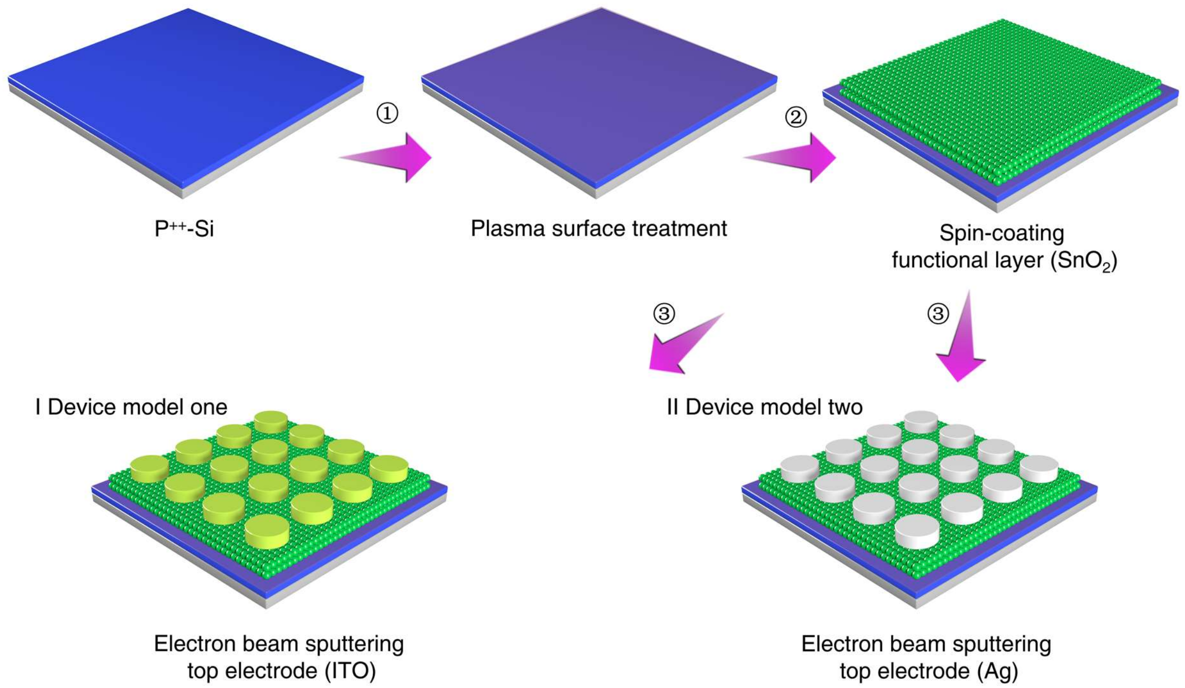

Figure S6. Schematic diagram of the device preparation process.

The detailed preparation process is as follows:

The $P^{++}Si$ substrate is cleaned by ultrasonication in deionized water for 15 minutes, followed by rinsing with ethanol and nitrogen blow-drying for later use. The plasma treatment is performed on the cleaned $P^{++}Si$ substrate surface.

The sample is ultrasonicated for 30 minutes, followed by spin coating at low speed (1000 rpm for 15 seconds) and high speed (3000 rpm for 45 seconds). The sample is heated for 5 minutes and the spin coating process is repeated. Such repetition is conducted for 5 times.

By covering with a mask and performing evaporation, two types of devices can be prepared. The same process is employed to prepare the upper electrodes using ITO and Ag, resulting in the final preparation of two types of devices.

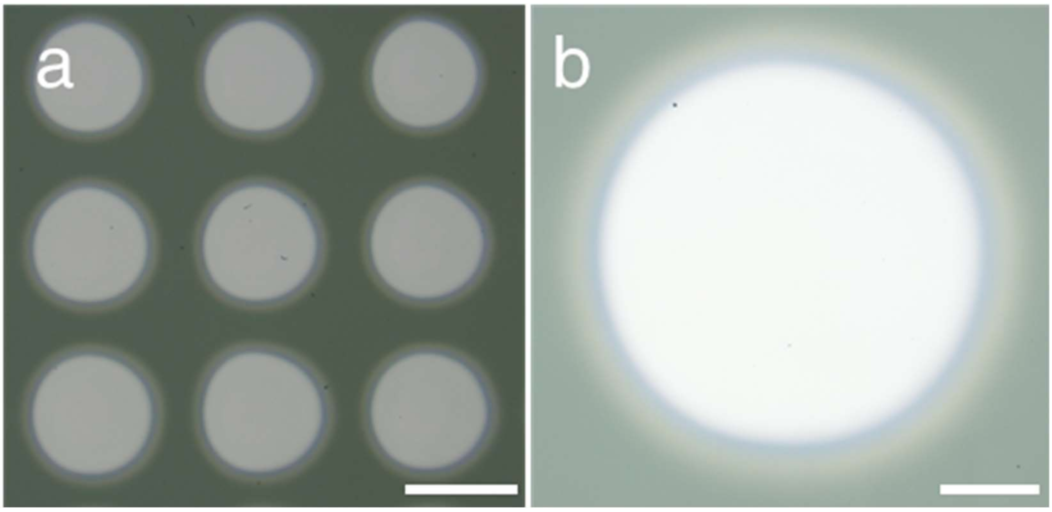

Figure S7. a) An optical photograph array illustrating Ag/SnO_2/p^{++}-Si memristors (scale: 400 μm), and b) a depiction of a single memristor device (scale: 100 μm).

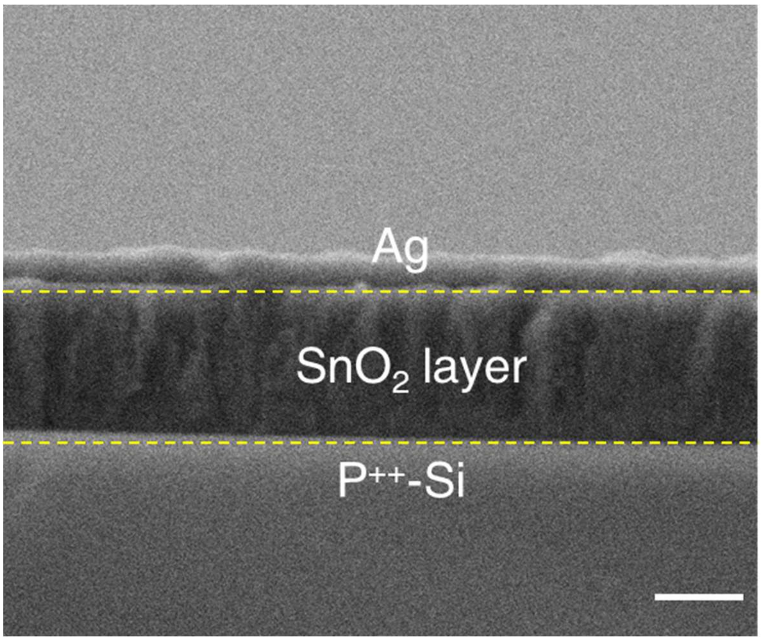

Figure S8. Cross-sectional scanning electron microscope (SEM) image, depicting the cross-section of the Ag/SnO_2/p++-Si memristor (scale: 100 nm).

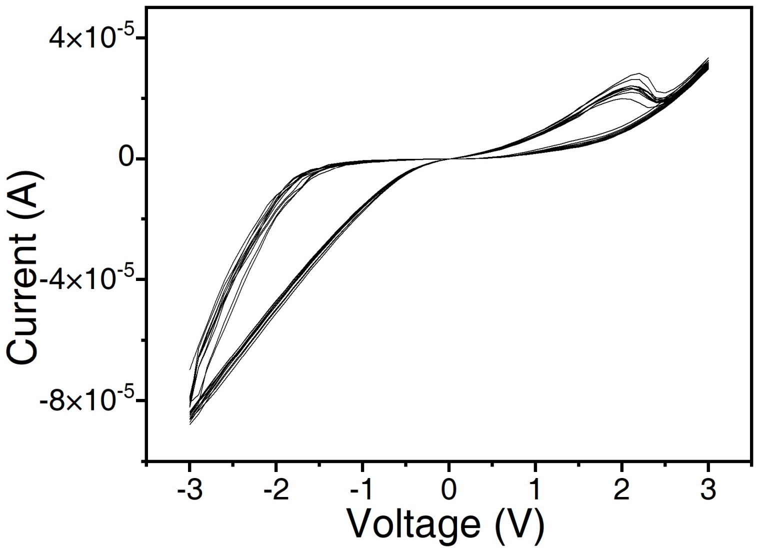

Figure S9. *I-V* curves of ITO/SnO_2/p^{++}-Si memristor scanned from -3 V to 3 V for ten cycles.

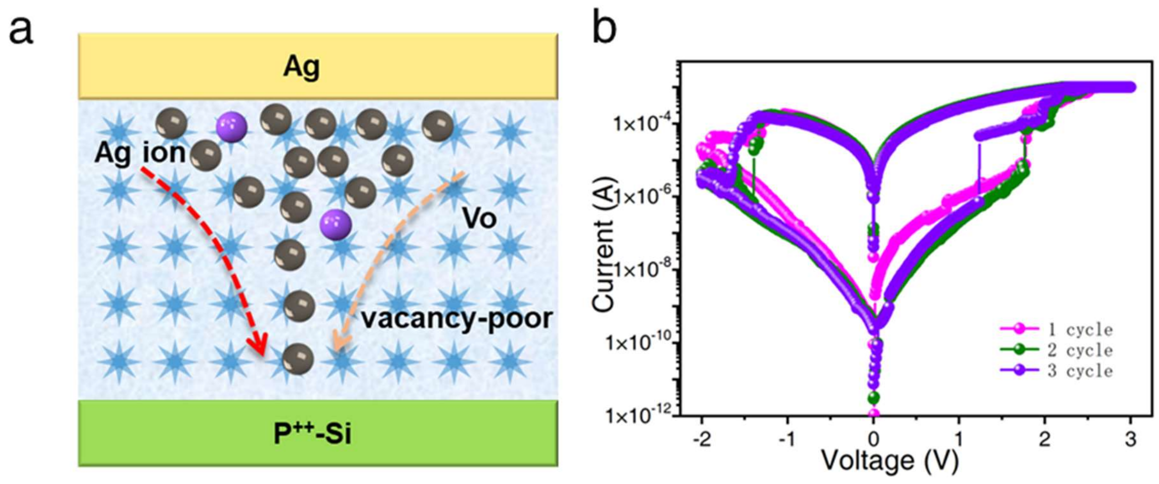

Figure S10. a) The conduction mechanism model for Ag/SnO_2/p^{++}-Si with vacancy-poor state and b) *I–V* characteristics.

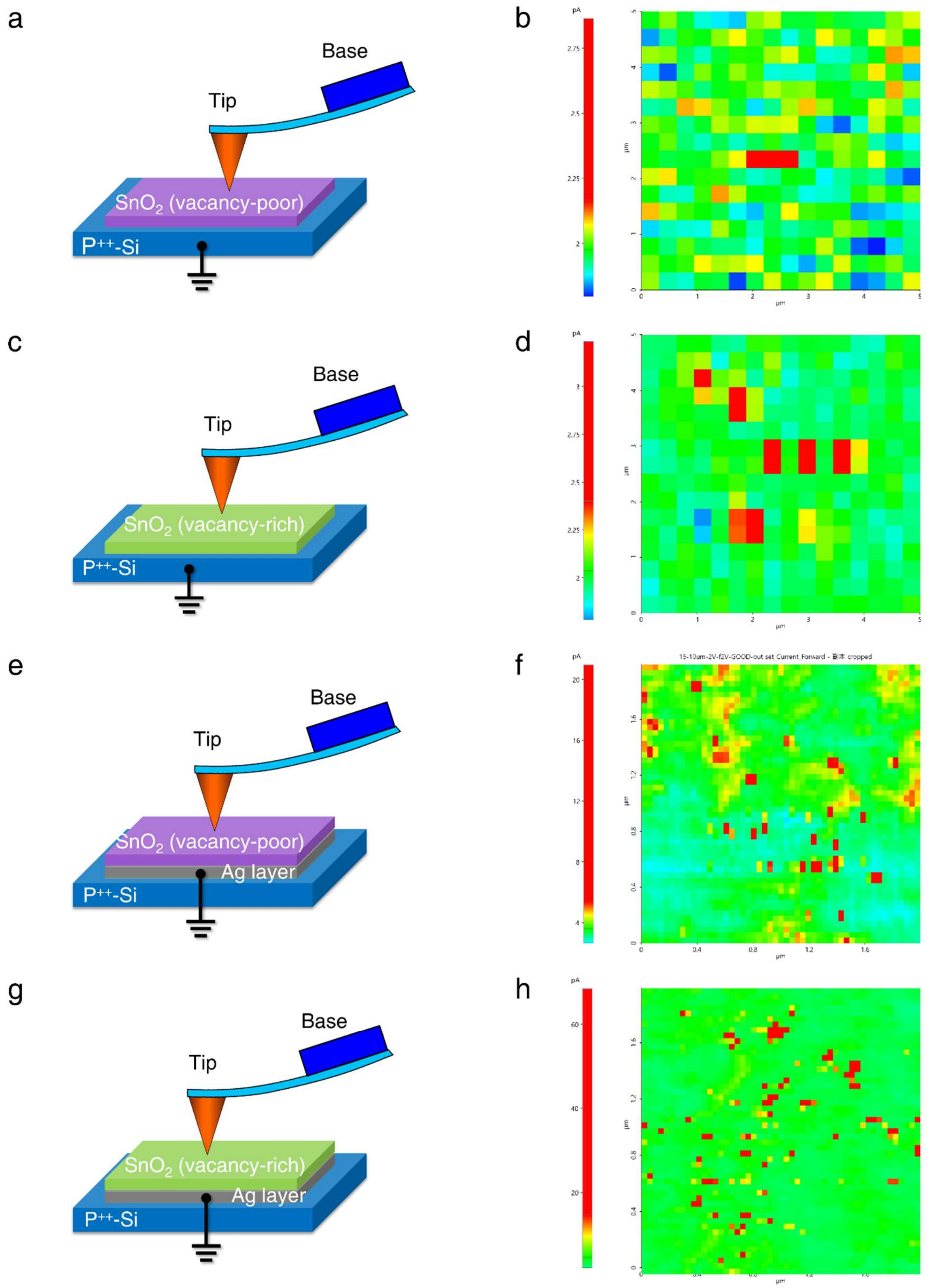

Figure S11. a) The device structure with SnO_2 (vacancy-poor)/P^{++}-Si and b) C-AFM mapping. c) The device structure with SnO_2 (vacancy-rich)/P^{++}-Si and d) C-AFM mapping. e) The device structure with SnO_2 (vacancy-poor)/Ag/P^{++}-Si and f) C-AFM mapping. g) The device structure with SnO_2 (vacancy-rich)/Ag/P^{++}-Si and h) C-AFM mapping.

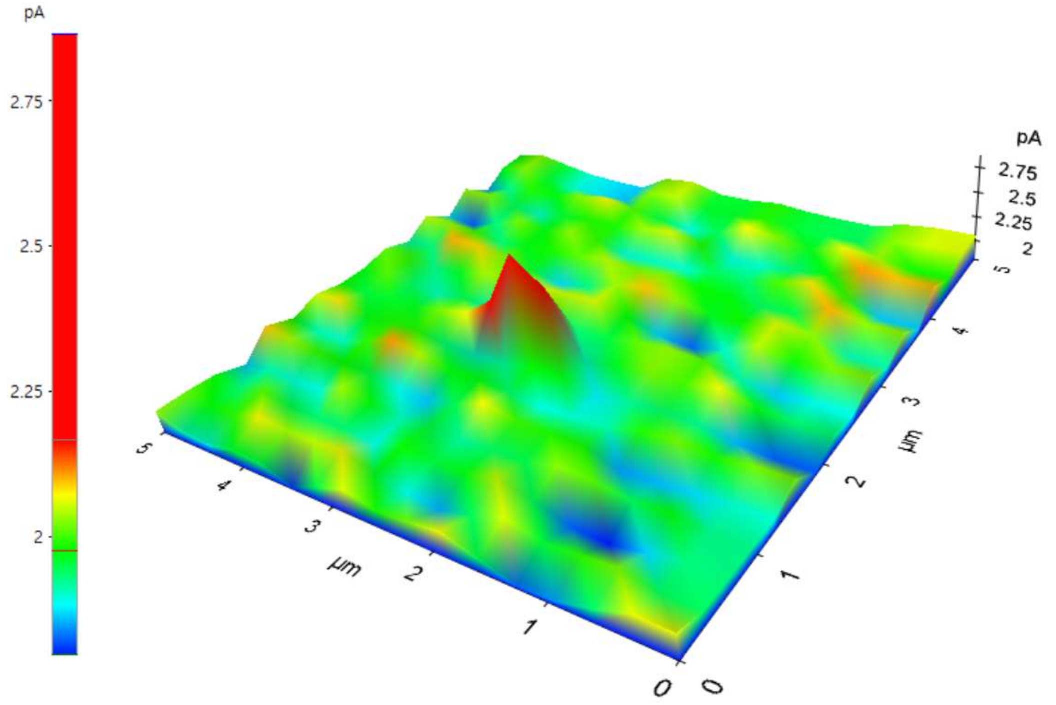

Figure S12. C-AFM mapping based on the device structure of SnO_2 (vacancy-poor)/P^{++}-Si.

Table S2. The optimized lattice constants of SnO_2 NFs and test result.

Model	*a* (Å)	*b* (Å)	*c* (Å)
Test model	4.7382	4.7382	3.1871
Calculation model	4 7372	4 .7372	31863

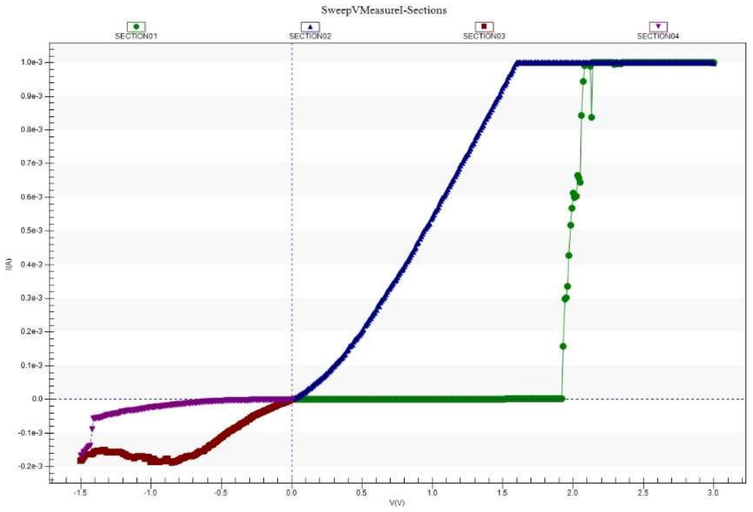

Figure S13. Typical *I–V* curve of $Ag/SnO_2/p^{++}$-Si memristor scanned from -1.5 V to 3 V.

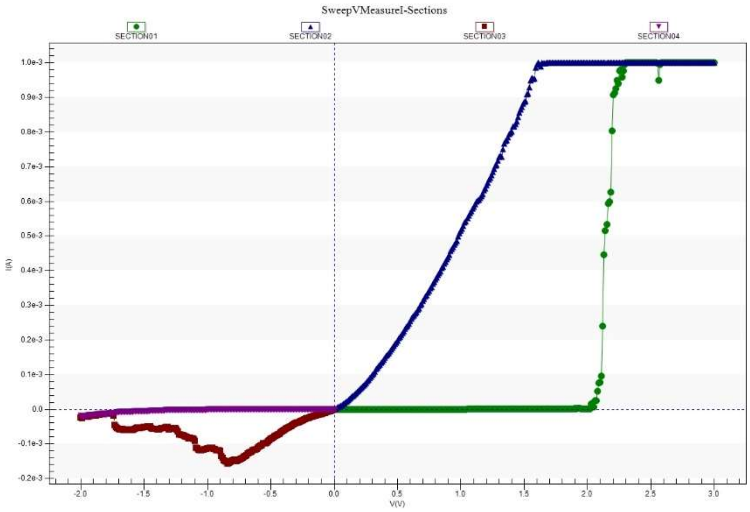

Figure S14. Typical *I–V* curve of Ag/SnO_2/p^{++}-Si memristor scanned from -2 V to 3 V.

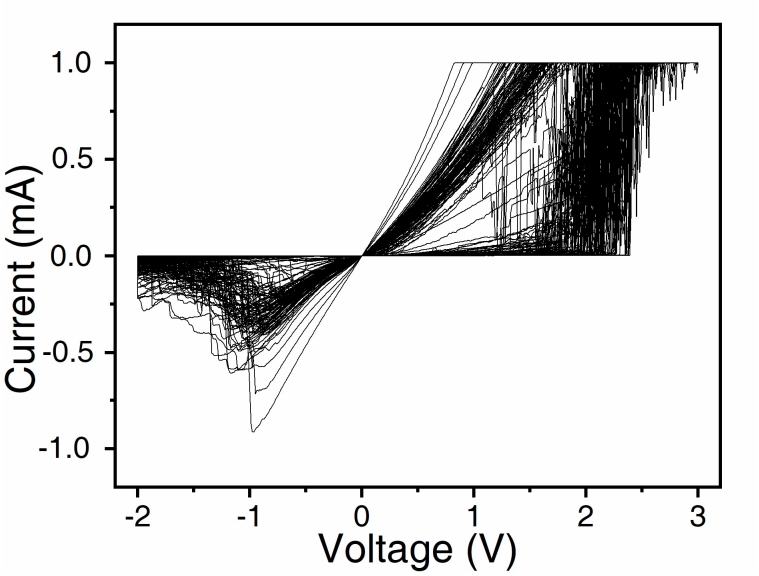

Figure S15. *I–V* curves of Ag/SnO_2/p^{++}-Si memristor scanned from -2 V to 3 V for 100 cycles.

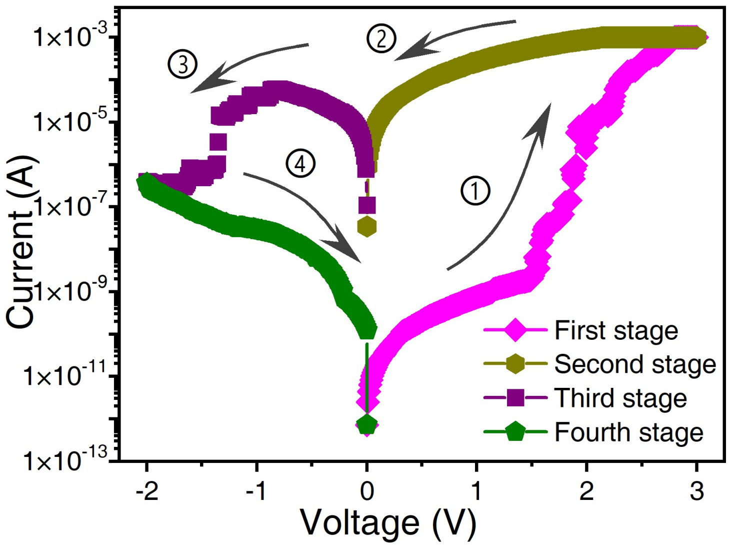

Figure S16. *I–V* characteristics of the Ag/SnO_2/p^{++}-Si device.

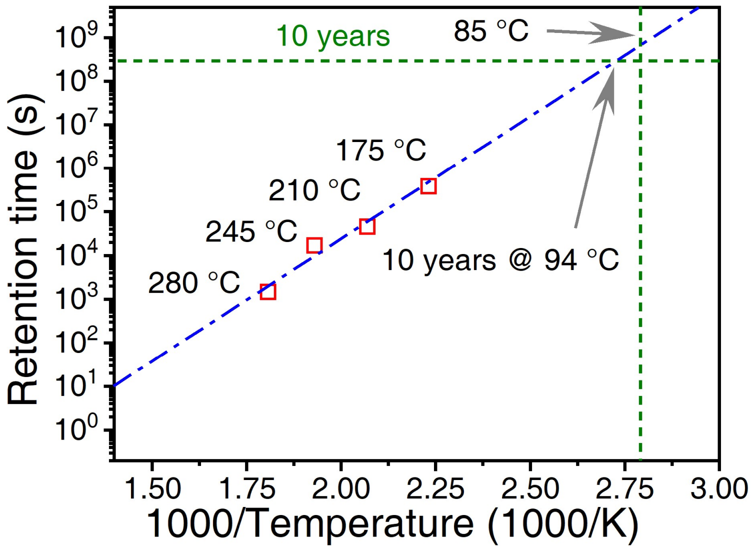

Figure S17. The temperature dependence of the retention time (s) following the Arrhenius relation for the Ag/SnO_2/p^{++}-Si device.[6] Baking temperatures were set at 175 °C, 210 °C, 245 °C, and 280 °C. A retention of 8.4869×10^8 s at 85 °C was extrapolated from the linear fit of measurement results.

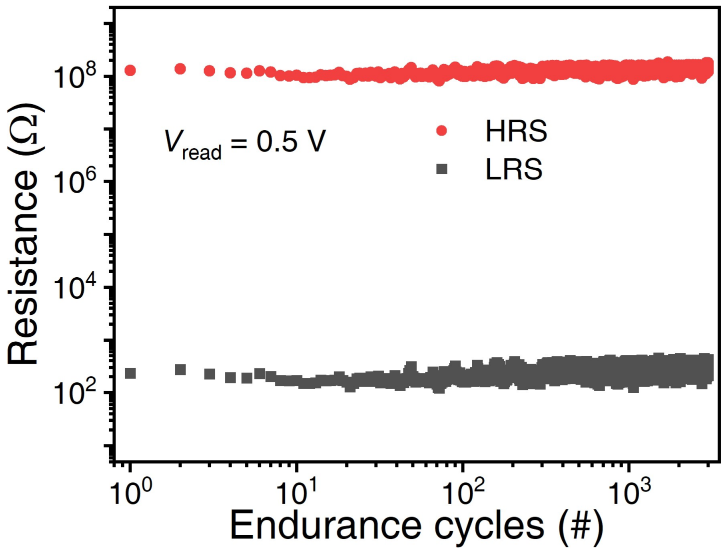

Figure S18. The endurance cycles of the devices were assessed through the application of SET and RESET pulse voltage scanning (±3 V, with a duration of 500 ns) and read pulse voltage (0.5 V, with a duration of 500 ns).

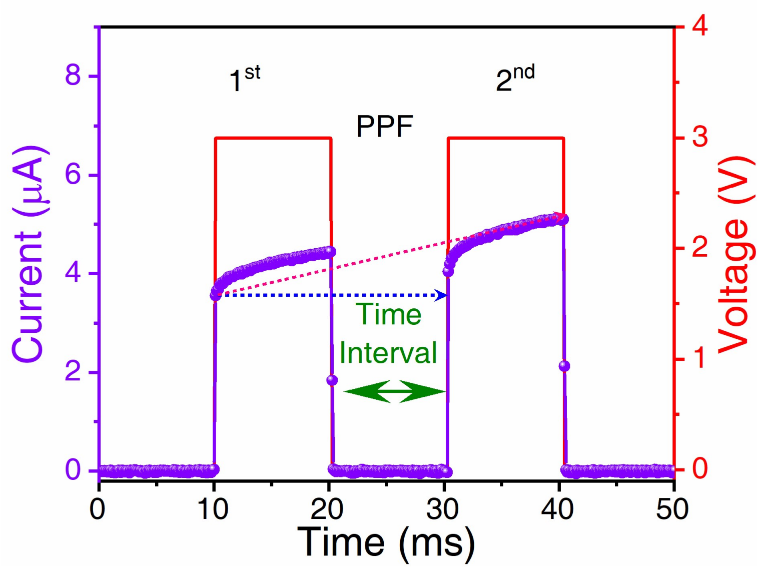

Figure S19. PPF test of Ag/SnO_2/p^{++}-Si device.

Table S3. Extracted fitting parameters for pattern recognition applications.

	alpha (P)	*alpha (D)*	G_{max}	G_{min}
SnO_2-based device	3.98e-06	-5.03e-06	4603 nS	884.42 nS

Table S4. Investigating the typical performance of emerging RRAM.

Category	Device structure	PPF	STM-to-LTM transition	Voltage-modulated plasticity	Multistate (Conductance Changes)	Switching Ratio	Retention Time (s)	Year	Ref.
SnO_2-Based Materials	Au/ SnO_2/PET	–	–	–	–	~10^5	–	2005	[7]
	Mo/SnS/SnO_{2-x}/ITO/Ag	–	–	–	–	33	–	2016	[8]
	Ag/SnO_2:Fe/Ag	–	√	√	<10 SET/RESET	~10^3	10^4	2017	[9]
	Pt/Ti/SiO_2/Si	–	–	–	–	~10^5	10^4	2019	[10]
	ITO/Bi: SnO_2/TiN	–	–	–	–	<10^2	10^4	2020	[11]
	Pt/SnO_2/Ti or Ag	–	–	–	–	10^2	10^4	2021	[12]
	Ti/Au/2D SnO_2/Au/Ti	–	√	√	50 SET/RESET	~10	10^4	2021	[13]
	Ag/TiO_x/F-doped SnO_2/FTO	–	–	–	50 SET/RESET	<10	–	2022	[14]
	Au/Mn-doped SnO_2/Si	–	–	–	<10 SET/RESET	<10^2	–	2023	[15]
	Ag/SnO_2/FTO	–	–	–	–	~10	1600	2023	[16]

	Au/MoS_2/Au	–	√	√	–	~10^5	1500	2018	[17]
	Ti/Ni/$MoTe_2$/Au/Ti	–	–	–	–	~10^6	10^4	2019	[18]
	Pt/Ta_2O_5/Ru	–	–	–	–	<10^5	10^4	2020	[19]
Other Materials	Au/$CuInP_2S_6$/Au	–	√	√	–	~10^3	3500	2021	[20]
	Ag/GQDs/TiO_x/FTO	–	–	–	–	<10^3	10^4	2021	[21]
	Ag/TiO_x/FTO	–	–	–	–	<10^2	10^4	2022	[22]
	Ag/SF/Au	–	√	√	–	10^4	1600	2023	[23]
**This Work**	Ag/SnO_2 NFs vacancy-rich/p^{++}-Si	√	√	√	60 SET/RESET	1.29×10^6	10^5	2023	–

References

[1] R. Garvie, J. Phys. Chem. C 1978, 82, 218.

[2] T. Nishinga, Book 2015.

[3] T. Nishinaga, *Handbook of crystal growth: fundamentals*, Elsevier, 2014.

[4] S. Das, V. Jayaraman, Prog. Mater Sci. 2015, 67, 161; H. S. P. Wong, H.-Y. Lee, S. Yu, Y.-S. Chen, Y. Wu, P.-S. Chen, B. Lee, F. T. Chen, M.-J. Tsai, Proceedings of the IEEE 2012, 100, 1951.

[5] M. Shao, J. Liu, W. Ding, J. Wang, F. Dong, J. Zhang, J. Mater. Chem. C 2020, 8, 487.

[6] D. Ielmini, R. Waser, Resistive switching: from fundamentals of nanoionic redox processes to memristive device applications, John Wiley & Sons, 2015. ISBN: 978-3-527-68093-1.

[7] M. Lyu, Y. Liu, Y. Zhi, C. Xiao, B. Gu, X. Hua, S. Fan, Y. Lin, W. Bai, W. Tong, Y. Zou, B. Pan, B. Ye, Y. Xie, J. Am. Chem. Soc. 2015, 137, 15043.

[8] Y.-J. Hong, T.-H. Wang, S.-Y. Wei, P. Chang, T.-R. Yew, Jpn. J. Appl. Phys. 2016, 55 060301.

[9] S. Trivedi, U. Joshi, Journal of Nano- and Electronic Physics 2017, 9, 101025.

[10] F. Mei, H. Shen, L. Li, G. Zang, Y. Shao, L. Liu, L. Lei, F. Huang, X. Lu, J. Zhu, J. Appl. Phys. 2019, 125, 074502.

[11] Y. Liu, C. Ye, K. C. Chang, L. Li, B. Jiang, C. Xia, L. Liu, X. Zhang, X. Liu, T. Xia, Z. Peng, G. Cao, G. Cheng, S. Ke, J. Wang, Small 2020, 16, 2004619.

[12] M. J. Yun, K. H. Kim, D. Bea, J. Jung, S. Kim, H.-D. Kim, J. Electr. Eng. Technol. 2021, 16, 1011.

[13]C. H. Huang, H. Chang, T. Y. Yang, Y. C. Wang, Y. L. Chueh, K. Nomura, ACS Appl. Mater. Interfaces 2021, 13, 52822.

[14]J. Li, G. Zhou, Y. Li, J. Chen, Y. Ge, Y. Mo, Y. Yang, X. Qian, W. Jiang, H. Liu, M. Guo, L. Wang, S. Duan, Artif. Intell. Rev. 2021, 55, 657.

[15]Z. Xu, T. Ji, S. Zhang, P. Guan, J. Elliott, T. Wan, C. Cazorla, D. Chu, Mater. Sci. Technol. 2023, 39, 1180.

[16]A. Kalateh, A. Jalali, M. J. Kamali Ashtiani, M. Mohammadimasoudi, H. Bastami, M. Mohseni, Sci. Rep. 2023, 13, 20036.

[17]V. K. Sangwan, H. S. Lee, H. Bergeron, I. Balla, M. E. Beck, K. S. Chen, M. C. Hersam, Nature 2018, 554, 500.

[18]F. Zhang, H. Zhang, S. Krylyuk, C. A. Milligan, Y. Zhu, D. Y. Zemlyanov, L. A. Bendersky, B. P. Burton, A. V. Davydov, J. Appenzeller, Nat. Mater. 2019, 18, 55.

[19]J. H. Yoon, J. Zhang, P. Lin, N. Upadhyay, P. Yan, Y. Liu, Q. Xia, J. J. Yang, Adv. Mater. 2020, 32, 1904599.

[20]J. Chen, C. Zhu, G. Cao, H. Liu, R. Bian, J. Wang, C. Li, J. Chen, Q. Fu, Q. Liu, P. Meng, W. Li, F. Liu, Z. Liu, Adv. Mater. 2021, 2104676.

[21]G. Zhou, B. Sun, X. Hu, L. Sun, Z. Zou, B. Xiao, W. Qiu, B. Wu, J. Li, J. Han, L. Liao, C. Xu, G. Xiao, L. Xiao, J. Cheng, S. Zheng, L. Wang, Q. Song, S. Duan, Adv. Sci. 2021, 8, 2003756.

[22]B. Yan, D. Kuang, W. Wang, Y. Wang, B. Sun, G. Zhou, Appl. Phys. Lett. 2022, 120, 253506.

[23]Z. Li, J. Wang, L. Xu, L. Wang, H. Shang, H. Ying, Y. Zhao, L. Wen, C. Guo, X. Zheng,

Adv. Mater. 2023, 2308843.
